# Charge Transfer in Patterned Bilayer Film of Ag/ZnS Composite by Magnetron Control Sputtering

**DOI:** 10.3390/molecules27123805

**Published:** 2022-06-13

**Authors:** Yongjun Zhang, Hailong Zhou, Lijun Liang

**Affiliations:** 1 Center for Advanced Optoelectronic Materials, Key Laboratory of Novel Materials for Sensor of Zhejiang Province, College of Materials and Environmental Engineering, Hangzhou Dianzi University, Hangzhou 310018, China; yjzhang@hdu.edu.cn; 2 Zhejiang Sunflux Electron Co., Ltd., Shaoxing 311266, China; hailongzhou@sunfluxeq.com; 3 College of Automation, Hangzhou Dianzi University, Hangzhou 310018, China

**Keywords:** Ag/ZnS bilayer film, nanocap arrays, SERS

## Abstract

Ordered heterojunction nanocap arrays composed of the bilayer film Ag/ZnS were prepared onto ordered two-dimensional polystyrene bead arrays by magnetron control sputtering, and the surface morphologies were tuned by changing the ZnS thickness. When the ZnS thickness varied from 10 to 30 nm with a Ag thickness of 5 nm, the roughness of the bilayer film Ag/ZnS increased obviously. The UV–VIS spectra showed the shifted LSPR peaks with ZnS thickness, which was attributed to the changes of the electron density as confirmed by Hall effect analysis. SERS observations confirmed the charge transfer process for the varied electromagnetic couplings when the ZnS thickness changed.

## 1. Introduction

In a basic physical view, all material properties are attributed to the behaviors of carriers, such as the movements and the interactions. When certain parameters of the materials are tuned, for example, the shapes, the chemical compositions, and the size, the movements and the interactions of the carriers are regulated, which results in varied properties, such as optical property, chemical property, and conducting property [1,2]. Carrier density is one of the key parameters for materials, affecting the movements and the interactions of the carriers. For most metals, the inherent carrier density around 10^22^ cm^−3^ can be maintained even when the metal size is decreased to nanometer scale, which indicates the difficulties in the metal property changes by the carrier density. In contrast, the carrier density of the semiconductor materials is under control in the range of 10^15^–10^21^ cm^−3^ by the doping technique, alloy process, and optical excitation [3,4]. When metal–semiconductor composites are fabricated, the bandgap distribution changes owing to the formation of the new Fermi level, which also affects the carrier movement over the interfaces in the heterojunction because of the significantly different work functions and carrier densities [5]. The regulated carrier movement over the interfaces in the heterojunction results in quite different electric and optical properties because of the one-way carrier movements [6]. According to the physical theory, the chemical bonds depend on the overlaps of the electron clouds, which show the electromagnetic interaction between the neighbor atoms. Therefore, the bonding energy changes when the electron density changes in the molecules. Surface-enhanced Raman scattering (SERS) is one of the modern techniques for accurate detection with molecular sensitivity, finding wide applications in various fields after years of developments [7,8,9]. For the fingerprint characteristics, SERS observations are usually applied to detect the pollution molecules by investigating the changes of certain peaks [10]. Despite the controversial enhancement mechanism of SERS until today, acceptable explanations for the sensing ability of SERS substrates include the electromagnetic enhancement (EM) mechanism and the chemical enhancement (CM) mechanism. In comparison with the EM mechanism caused by localized surface plasmon resonance (LSPR), the CM mechanism is mainly rooted in the charge transfer process through the bonds between the sensing substrate and the linked molecules [11,12,13]. For composites composed of plasmonic metals and semiconductors, the one-way charge transfer from metal to semiconductor means possible in situ research on the surface or interface of the heterojunctions. On the other hand, changes on the surface or interface of the heterojunctions also have effects on the charge transfer process, which shows obvious changes in SERS observations.

As one of the wide and direct bandgap semiconductors, ZnS shows good optical performance with a bandgap energy of 3.6 eV, which finds wide applications in light-emitting diodes (LEDs), electroluminescence, flat panel displays, infrared windows, sensors, lasers, and biodevices [14,15,16]. To improve their optical performance, ZnS materials of different microstructures are prepared to regulate the behaviors of carriers, such as ZnS nanostructures of different shapes, components, dopants, and heterojunctions. The charge transfer properties related to the interfaces in the metal–ZnS heterojunctions are vital for applications in various fields, which can be changed by controlling the ZnS thickness [17,18]. The SERS peaks of the probe molecule are sensitive to the charge transfer process, which indicates that SERS is promising in exploring the interface charge transfer process. In this work, a Ag/ZnS bilayer film is deposited onto two-dimensional (2D) polystyrene (PS) bead arrays by magnetron control sputtering. SERS observations are conducted to characterize the dependence of the charge transfer on ZnS thickness, which is confirmed by UV–VIS spectroscopy and Hall effect measurements.

## 2. Experiments

4-Mercaptobenzoic acid (4-MBA) powder was bought from Sigma-Aldrich Co. Ltd. (Beijing, China). A suspension of the polystyrene bead at 500 nm (10 wt%) was bought from Duke Co. Ltd. Palo Alto, CA, USA, and the size deviation of the polystyrene beads was below 10%. Ag and ZnS targets were bought from the Beijing TIANRY Science and Technology Developing Center (Beijing, China). Silicon wafers were bought from Hefei Kejing Materials Technology Co. Ltd. (Hefei, China). A polystyrene bead monolayer film was prepared by self-assembly method, as reported in our previous works [19,20]. In brief, the silicon wafer was boiled in a boiling solution of ammonia, hydrogen peroxide, and deionized water (volume ratio of 1:2:6) for 20 min, which resulted in a hydrophilic surface favorite for the self-assembly process of the polystyrene beads. The polystyrene bead suspension and absolute ethanol with a volume ratio of 10:7 were mixed together by sonication for 5 min. Then, a dense monolayer composed of the polystyrene beads formed on the deionized water surface because of the surface tension when the mixed solution was applied to the deionized water. The ordered monolayer of the polystyrene beads was picked up by the cleaned silicon wafer, which was used as the substrate for Ag and ZnS deposition in the magnetron control sputtering system. For the sputtering process, the base pressure was 2 × 10^−6^ Pa, the reaction pressure was 0.6 Pa, and the distance between the target and the substrate was 10 cm. A sputtering power of 20 W was applied for ZnS deposition, and 10 W for Ag deposition. A scanning electron microscope (SEM, 15 kV, JEOL 6500F) and a transmission electron microscope (TEM, 200 keV, JEM-2100H) were used to characterize the surface morphologies and microstructures. Hall effect measurement was performed on the Hall effect detector mode Lakeshore, 775 HMS Matrix. UV–VIS spectrum measurement was performed on a Shimadzu UV-3600 spectrophotometer (Kyoto, Japan). Raman spectrum measurement was performed on Renishaw Raman, and the spectral resolution was 1 cm^−1^ (London, UK, 2000 confocal microscope spectrometer).

## 3. Results and Discussions

**Figure 1:**
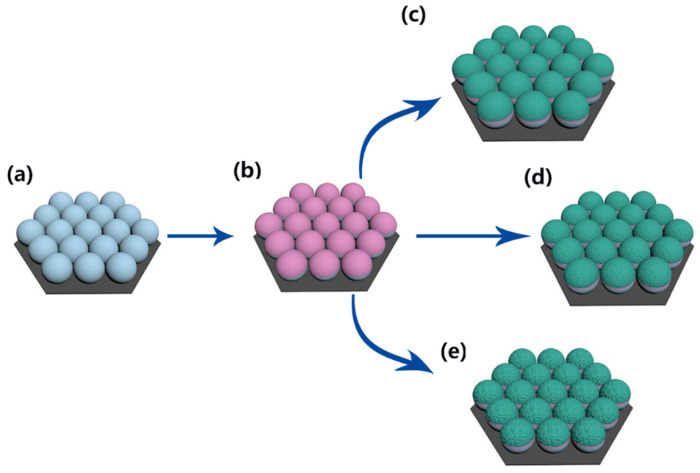
Schematic illustration of Ag and Ag/ZnS film deposition onto a PS array. (**a**) Ordered PS bead array by self-assembly technique; (**b**) Ag film deposition onto PS bead array; (**c**–**e**) ZnS film deposition with different time.

**Figure 2:**
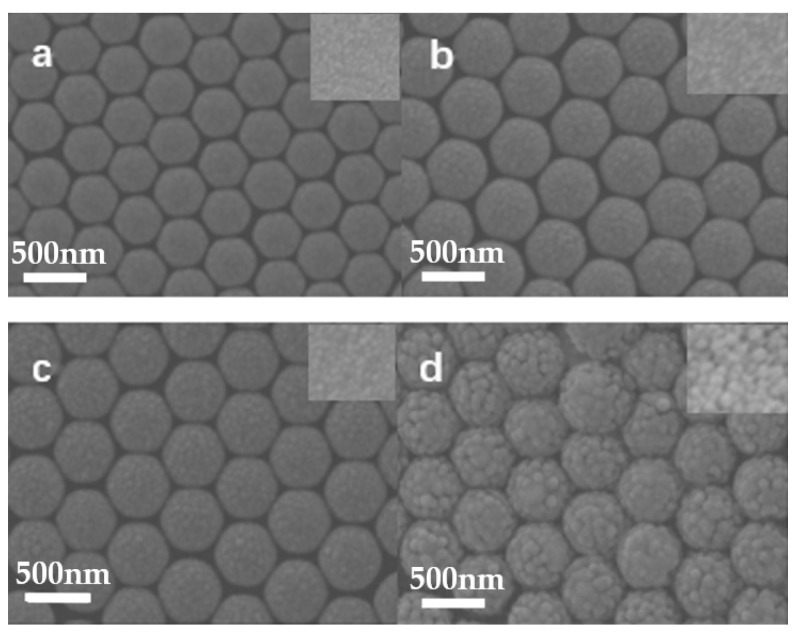
SEM images of Ag/ZnS ((**a**) 0 min, (**b**) 5 min, (**c**) 10 min, (**d**) 15 min) films on a PS array.

**Figure 3:**
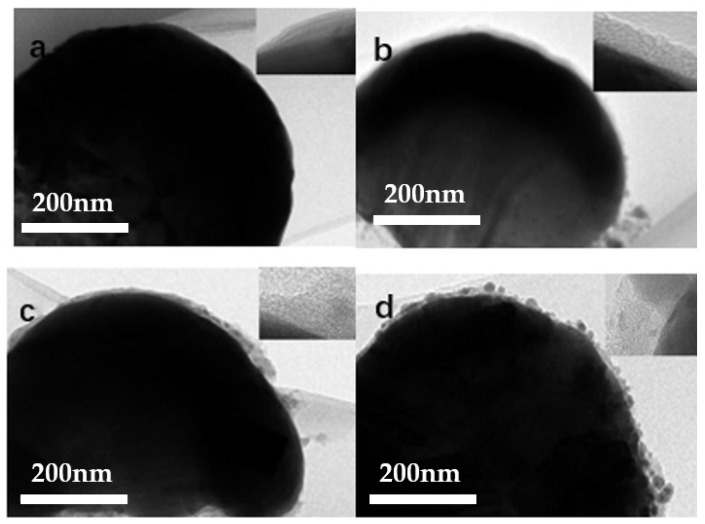
TEM images of Ag/ZnS ((**a**) 0 min, (**b**) 5 min, (**c**) 10 min, (**d**) 15 min) films on PS beads.

As shown in the illustration (Figure 1), Ag at 5 nm is deposited onto a PS array first, which is followed by ZnS deposition. This experimental setup can tune ZnS thickness and Ag thickness by changing the sputtering time separately. ZnS thickness shows effects on the surface morphologies, which depends on the sputtering time in our experiments. When the ZnS growth time is short, the surface roughness is similar to the roughness of the Ag film. When the ZnS deposition time is prolonged, a large roughness of the ZnS film is observed due to ZnS growth. SEM images in Figure 2 confirm that the surface morphologies depend on the ZnS growth time. When a Ag film with a thickness of 5 nm is deposited onto a PS array, the curved Ag film forms on PS beads. When ZnS is deposited onto a Ag film for 5 min, the film surface shows no significant changes and the ZnS particle size is around 10 nm. When the film growth time increase from 5 to 10 min, the ZnS particle size increases to 15 nm, as shown in the inset image. When film growth time increases from 10 to 15 min, the particle size increases to 50 nm, as shown in the inset image. TEM images in Figure 3 confirm the curved film induced by the curved surfaces of PS beads. For the growth time of 5 min, the TEM image shows a clear growth of the ZnS layer with the smooth surface. When the growth time increases from 5 to 10 min and, finally, to 15 min, ZnS nanoparticle formation and growth are observed, in agreement with the SEM image. The HRTEM images in the insets show the existence of Ag nanoparticles in the ZnS layer, which indicates Ag diffusions into the ZnS layer during ZnS growth. For the ZnS growth process by magnetron control sputtering, ZnS clusters release energy when they land on the film surface. The energy release accelerates Ag diffusion, which results in Ag nanoparticle formation in the ZnS layer, similar to observations reported for other composites [21,22].

According to the Drude model, the plasmon frequency (ω_p_) shows the dependence on the electron density as expressed by Equations (1) and (2), which means that the changes in the free carrier concentrations results in the shift of the LSPR peak, where N_e_ stands for the free electron density, m for the effective mass of the electron, and ε for the dielectric function [23].

ω_p_ = (N_e_e^2^/mε)^1/2^ (1)

ω_p_ ∝ λ^−1^ (2)

**Figure 4:**
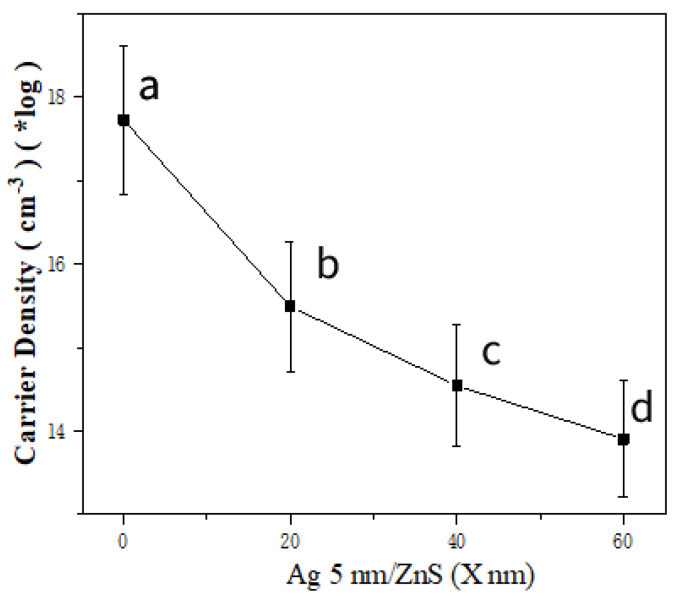
The dependence of electron density on ZnS thickness for Ag/ZnS (a. 0 min; b. 5 min; c. 10 min; d. 15 min) films on PS beads.

Based on the Drude model, it is seen that in semiconductors, when the magnetic field is applied to the current direction vertically, the electrons change their movements because of the Lorentz forces, resulting in the lateral voltage stabilizing the whole system, which is known as Hall effect. For the Hall effect, the Hall coefficient is in the inverse proportion to the carrier density expressed by the function K = 1/(n × q), where n is the carrier density. The Hall effect is employed to quantitatively analyze the dependence of the carrier density on ZnS thickness in our system. In our Ag–ZnS heterojunction structure, Ag works as the electron reservoir, which is reduced with the increase in ZnS thickness. For the Ag film on a PS array, the electron density is around 10^20^ lower than 10^22^ for bulk Ag, because the curved Ag film on each PS bead is isolated and composed of some Ag nanoparticles. The uncontinuous Ag film disturbs the electron movement and decreases the electron supply, which leads to a decreased electron density, as shown in Figure 4. When the ZnS thickness increases as the growth time increases, the measured electron density decreases significantly because the intrinsic electron density in ZnS is extremely low in comparison with metal Ag, and the resistance of the ZnS layer decreases the electron supply from metal Ag.

**Figure 5:**
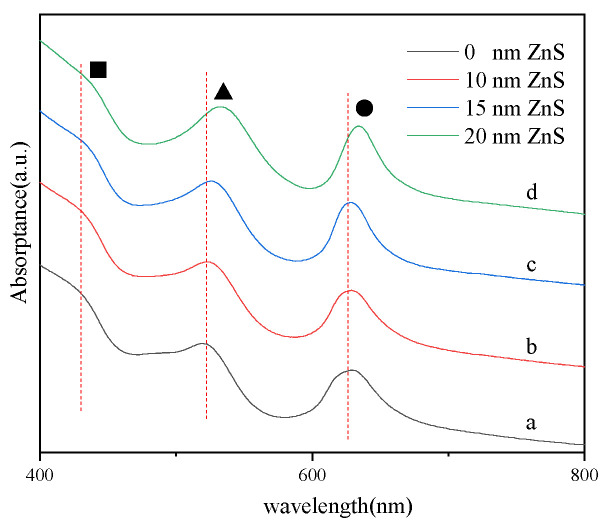
UV–VIS absorption spectra for Ag/ZnS (a. 0 min b. 5 min; c. 10 min; d. 15 min) films on PS beads.

The UV–VIS absorption spectra in Figure 5 show two peaks labeled with a triangle and a star, which are from the LSPR of the Ag–ZnS array. Three absorption bands are observed, the band around 437 nm, the band around 533 nm, and the band around 633 nm. The band around 437 nm labeled with a square is assigned to the LSPR of Ag. With the increasing thickness of ZnS, the peak assigned to the LSPR of Ag is red-shifted due to the refractive index effect of ZnS. The band around 533 nm labeled with a triangle and the band around 633 nm labeled with a dot are from the LSPR of the Ag–ZnS array, which are due to the interactions between the neighbor Ag nanocaps. When the ZnS thickness increases, the carrier density decreases due to the increased dielectrics, which leads to the significantly red-shifted absorption band, consistent with the Hall effect observations.

**Figure 6:**
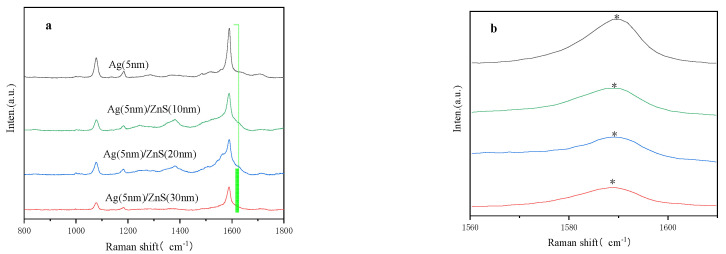
SERS observation of 4–MBA linked to the substrate Ag/ZnS (0–15 min) films on a PS bead array with different ZnS thicknesses (**a**). The magnification image shows the shift of the peak around 1588 cm^−1^ when the ZnS thickness changes, which is labelled by the star (**b**).

The different samples are chosen to evaluate their potential usage as SERS-active substrates, and a series of SERS signals generated by 4-MBA (10^−3^ M) probe molecules are allowed to be absorbed on. The spectra are collected with HeCd laser irradiation (633 nm). The laser power is set as 17 mW, and the laser power attenuation is set as 1% with a one-time accumulation of 10 s exposure time. The significant SERS signals are observed for all samples, as shown in Figure 6. In comparison with the pure Ag nanocap array, the SERS signals show a continuous decrease for the samples with increased ZnS film thickness.

To quantitatively evaluate the enhancement ability for the different Ag/ZnS substrates, the enhancement factors (EF) are calculated by the expression EF = (*I_SERS_* × *N_bulk_*)/(*I_bulk_* × *N_SERS_*) [24,25]. *I_SERS_* represents the SERS signals from 4-MBA absorbed on the Ag/ZnS substrate, and *I_bulk_* represents the SERS signals from 4-MBA powders. *N_SERS_* is the number of 4-MBA molecules absorbed on the Ag/ZnS substrates. *N_bulk_* is the number of 4-MBA powders. When the peak at 1588 cm^−1^ is chosen for EF calculation, the EF is 1.37 × 10^9^ for the Ag substrates, 1.60 × 10^7^ for the Ag/ZnS substrate with a ZnS growth time of 5 min, 7.48 × 10^6^ for the Ag/ZnS substrate with a ZnS growth time of 10 min, and 1.28 × 10^6^ for the Ag/ZnS substrate with a ZnS growth time of 15 min. The enhancement mechanism shows the evolution from electromagnetic field coupling to chemical enhancement, because the increased ZnS thickness results in the reduced process of the electromagnetic field coupling, which leads to the relative significances of the charge transfer process.

## 4. Conclusions

The bilayer film Ag/ZnS was deposited onto ordered 2D PS bead arrays with various ZnS thicknesses, and the different surface morphologies were observed for ZnS thickness changes. When the ZnS deposition time varied from 5 to 15 min with a Ag thickness of 5 nm, the surface morphologies of the bilayer film Ag/ZnS showed that the nanoparticle sizes increased from 10 to 15 nm and, finally, to 50 nm. At the same time, the thickness changes of the ZnS film resulted in a decreased electron density, as confirmed by Hall effect observation, which led to the shifted LSPR peaks and the changed enhancement mechanism for SERS observations from electromagnetic coupling to carrier transfer.
